# NEUROD1 predicts better prognosis in pancreatic cancer revealed by a TILs-based prognostic signature

**DOI:** 10.3389/fphar.2022.1025921

**Published:** 2022-10-13

**Authors:** Zhiyang Jiang, Jiadong Pan, Jiahui Lu, Jie Mei, Rui Xu, Dandan Xia, Xuejing Yang, Huiyu Wang, Chaoying Liu, Junying Xu, Junli Ding

**Affiliations:** ^1^ Department of General Surgery, Affiliated Wuxi People’s Hospital of Nanjing Medical University, Wuxi, China; ^2^ Department of Gastroenterology, Affiliated Wuxi People’s Hospital of Nanjing Medical University, Wuxi, China; ^3^ Department of Oncology, Affiliated Wuxi People’s Hospital of Nanjing Medical University, Wuxi, China; ^4^ The First College of Clinical Medicine of Nanjing Medical University, Nanjing, China

**Keywords:** TILs, prognosis, TCGA, NEUROD1, pancreatic cancer

## Abstract

It has been well-defined that tumor-infiltrating lymphocytes (TILs) play critical roles in pancreatic cancer (PaCa) progression. This research aimed to comprehensively explore the composition of TILs in PaCa and their potential clinical significance. A total of 178 samples from the TCGA and 63 samples from the GSE57495 dataset were enrolled in our study. ImmuCellAI was applied to calculate the infiltrating abundance of 24 immune cell types in PaCa and further survival analysis revealed the prognostic values of TILs in PaCa. Moreover, the Hallmark enticement analysis of differentially expressed genes (DEGs) between low- and high-risk groups was performed as well. Immunohistochemistry staining was used to evaluate NEUROD1 expression. As result, different kinds of TILs had distinct infiltrating features. In addition, Specific TILs subsets had notable prognostic values in PaCa. We further established a 6-TILs signature to assess the prognosis of PaCa patients. Kaplan-Meier and Cox regression analyses both suggested the significant prognostic value of the signature in PaCa. Based on the prognostic signature, we screened a great deal of potential prognostic biomarkers and successfully validated NEUROD1 as a novel prognostic biomarker in PaCa. Overall, the current study illuminated the immune cells infiltrating the landscape in PaCa and identified a TILs-dependent signature and NEUROD1 for prognostic prediction in PaCa patients.

## Introduction

Pancreatic cancer (PaCa) is one of the most fatal cancerous diseases worldwide, which is characterized by dreadful aggressiveness and poor prognosis. PaCa mainly affects older adults, and as the percentage of adults > 65 years old increases with the aging of the baby boomers, the prevalence is expected to rise over the coming decade (Winer and Dotan, 2021). The early diagnosis of PaCa is difficult due to the obscure symptoms, and its morbidity and mortality have been increasing significantly in recent years (Siegel et al., 2022). According to the newest statistical data published by the American Cancer Society, there will be about 62,210 new PaCa cases and nearly 50,000 cancer-causing deaths in 2022 in the United States (Siegel et al., 2022). Thus, further exploration of the molecular mechanisms underlying oncogenesis and development is essential to control the threat of PaCa.

With the constant development of advanced therapeutic strategies, immunotherapy is becoming a novel promising hotspot in the field of PaCa treatment (Foley et al., 2016). The tumor immune microenvironment, which contains extracellular matrix, fibroblasts, endothelial cells, and multiple immune cells, plays a critical role in determining response to immunotherapy (Dougan, 2017). Increasing evidence reveals that tumor progression is significantly affected by the host immune response, which is represented by the abundance of tumor-infiltrating lymphocytes (TILs) (Tahkola et al., 2018; Stenzel et al., 2020; Cai et al., 2021). However, the profiles and clinical significance of TILs in PaCa have not been well defined.

In the past decades, high-throughput sequencing technologies, including RNA sequencing (RNA-seq) and microarray, produce massive transcriptome data, which makes estimating the abundance of TILs by gene expression data possible. Several classic algorithms, including CIBERSORT (Newman et al., 2015), xCell (Aran et al., 2017), TIMER (Li et al., 2017), EPIC (Racle et al., 2017), and MCPcounter (Becht et al., 2016) have been established to calculate immune cell abundance based on transcriptome data of tumor samples. Encouragingly, Miao *et al.* developed a highly accurate method named ImmuCellAI to estimate the infiltrating levels of immune cells from transcriptome data (Miao et al., 2020). ImmuCellAI expands the scope of infiltrating assessment of more T cell subsets, such as regulatory T cell (Treg), cytotoxic T cell (Tc), and exhausted T cell (Tex). In addition, compared with other methods, ImmuCellAI has the highest consistency with flow cytometry results for most immune cells.

In this research, based on the ImmuCellAI approach and PaCa transcriptome data from TCGA and GEO datasets, we conducted an in-depth analysis of the TILs in PaCa samples. As a result, six kinds of immune cells, including nTreg, T helper 1 cell (Th1), Th17, dendritic cell (DC), CD4^+^ T cell, and CD8^+^ T cell, were developed as a promising predictive signature for prognostic assessment for PaCa patients. Based on the novel signature, we screened a great deal of potential prognostic biomarkers and successfully validated NEUROD1 as a novel prognostic biomarker in PaCa.

## Materials and methods

### Data acquisition

Normalized RNA-seq data and clinical information of PaCa samples were downloaded from the UCSC Xena website (https://xenabrowser.net/datapages/). Patients with missing or insufficient data were excluded from this research. Finally, 178 tumor samples with immune cell infiltrating data were reserved for further analysis. To validate the established prognostic signature, the gene expression data normalized by the RMA algorithm and clinical information of GSE57495 was obtained from the GEO database (https://www.ncbi.nlm.nih.gov/geo/query/acc.cgi?acc=GSE57495) (Chen et al., 2015), a total of 63 PaCa samples were included. The detailed clinic-pathological characteristics of PaCa patients from two datasets were exhibited in Table 1.

**TABLE 1:** Baseline characteristics of PaCa patients from the TCGA and GSE57495 datasets.

Characteristics	TCGA-PaCa		GSE57495	
	Cases	Proportion	Cases	Proportion
**Gender**				
Female	80	44.94%	30	47.62%
Male	98	55.06%	33	52.38%
Age				
≤60	59	33.15%	-	-
>60	119	66.85%	-	-
**Subdivision**				
Body	14	7.87%	-	-
Head	138	77.53%	-	-
Tail	15	8.43%	-	-
Other	11	6.18%	-	-
**Histology grade**				
Well-Differentiated	30	16.85%	6	9.52%
Moderate-Differentiated	96	53.93%	35	55.56%
Poor-Differentiated	50	28.09%	18	28.57%
Unknown	2	1.12%	4	6.35%
**T stage**				
T1	7	3.93%	2	3.17%
T2	24	13.48%	14	22.22%
T3	142	79.78%	47	74.60%
T4	3	1.69%	-	-
Unknown	2	1.12%	-	-
**N stage**				
N0	50	28.09%	30	47.62%
N1	123	69.10%	32	50.79%
Unknown	5	2.81%	1	1.59%
**M stage**				
M0	80	44.94%	-	-
M1	5	2.81%	-	-
Unknown	93	52.25%	-	-
**TNM stage**				
TNM 1	21	11.80%	13	20.63%
TNM 2	146	82.02%	50	79.37%
TNM 3	4	2.25%	-	-
TNM 4	5	2.81%	-	-
Unknown	5	2.81%	-	-
**OS status**				
Alive	85	47.75%	21	33.33%
Dead	93	52.25%	42	66.67%

### Immune infiltration analysis

ImmuCellAI (http://bioinfo.life.hust.edu.cn/web/ImmuCellAI/) is an emerging tool to estimate the abundance of 24 immune cells based on gene expression profile (Miao et al., 2020). Infiltrating data of TILs corresponding to TCGA-PaCa samples was downloaded from the ImmuCellAI website. Besides, TILs abundance of PaCa samples from the GSE57495 dataset was predicted by the “Analysis” module of ImmuCellAI by uploading gene expression data.

### LASSO Cox analysis

In survival analysis, the overall survival (OS) event was set as the end point of observation. To establish a TILs-dependent prognostic signature, univariate Cox regression was first used to screen the prognostic values of 24 TILs abundance. The least absolute shrinkage and selection operator (LASSO) Cox regression model was then applied for the further selection of prognostic TILs. The R package “glmnet” was used for LASSO analysis and for establishing the final model. Risk-score was calculated using a combination of the infiltrating abundance of TILs and regression coefficients. Kaplan-Meier curves and log-rank analysis were used to identify survival differences by setting the cut-off value at the median risk-score. Receiver operating characteristic (ROC) curves of the risk-score were generated using the R package “ROCR” to assess the prognostic accuracy of risk signature. Univariate and multivariable Cox analyses were used to study the independent prognostic value of risk-score combined with other clinic-pathological characteristics. At last, the GSE57495 dataset was used as the validation cohort to certify the effect of the risk-score signature.

### Identification of differentially expressed genes

R language was applied to screen differentially expressed genes (DEGs) between low-risk and high-risk samples using the R package “limma”. The thresholds of extracting DEGs were as follows: |log2(fold change (FC))| > 1, and *p* < 0.05. Volcano plots were drawn using the SangerBox tool (Shen et al., 2022).

### Function enrichment analysis

For gene set enrichment analysis, we downloaded the h.all.v7.4.symbols.gmt subclass from the Molecular Signatures Database (Liberzon et al., 2011), which was used as the background. The enrichment analysis was performed using the R package “clusterProfiler”. To obtain the results of gene set enrichment, the minimum gene set was set to 5 and the maximum gene was set to 5,000. The top 5 terms were exhibited as results.

### Collection of PaCa samples

The tumor tissue microarray of PaCa (TMA, HPanA150Su01) was purchased from Outdo BioTech (Shanghai, China). The HPanA150Su01 cohort contained 90 PaCa and 60 para-tumor samples. Detailed clinic-pathological information on the TMA and follow-up data were provided by Outdo BioTech. Ethical approval was granted by the Clinical Research Ethics Committee in Outdo Biotech (Shanghai, China).

### Immunohistochemistry staining and semi-quantitative assessment

Immunohistochemistry (IHC) staining was conducted on the HPanA150Su01 TMA according to the standardized procedures. The TMA was washed with xylene for three 5-min. The sections were rehydrated by successive washes in 100, 90 and 70% graded ethanol. Hydrogen peroxidase was used to block endogenous peroxidase activity for 20 min. The antigen retrieval solution is Ethylene Diamine Tetraacetic Acid. The primary antibody used in our research was anti-NEUROD1 (1:200 dilution, Cat. 12081-1-AP, ProteinTech). Antibody staining was visualized with DAB and hematoxylin counterstain, and stained TMA was captured using Aperio Digital Pathology Slide Scanners. During the IHC staining processes, two samples were separated, thus a total of 88 samples were included in further analysis. The stained TMA was independently evaluated by two pathologists. Expression levels of NEUROD1 in tumor cells were semi-quantitatively assessed by estimating the immunoreactivity score (IRS) (Mei et al., 2020; Mei et al., 2021). Briefly, the percentage of positively stained cells was scored as 0–4: 0 (< 5%), 1 (6–25%), 2 (26–50%), 3 (51–75%) and 4 (>75%). The staining intensity was scored as 0–3: 0 (negative), 1 (weak), 2 (moderate), and 3 (strong). The IRS equals the percentages of positive cells multiplied by staining intensity. Samples with NEUROD1 IRS ≥ 2 were deemed to be the high-expression group, and the others were deemed to be the low--expression group.

### Statistical analysis

R 4.0.2, SPSS 22.0, and GraphPad Prism 8.0 were applied as main tools for the statistical analysis and figures exhibition. The LASSO Cox regression model was employed for the further selection of prognostic TILs by “glmnet”. Kaplan-Meier survival plots were generated with survival curves compared by log-rank test. The Chi-square test was used to evaluate differences in clinic-pathological variables between groups with different risks or NEUROD1 expression. Univariate and multivariate Cox regression models were used to calculate hazard ratio (HR) of risk-score and other clinic-pathological variables for OS. For DEGs screening, R language was applied using the R package “limma”. For all analyses, differences were considered statistically significant when *p*-value < 0.05.

## Results

### The distribution and prognostic value of TILs in PaCa

**FIGURE 1:**
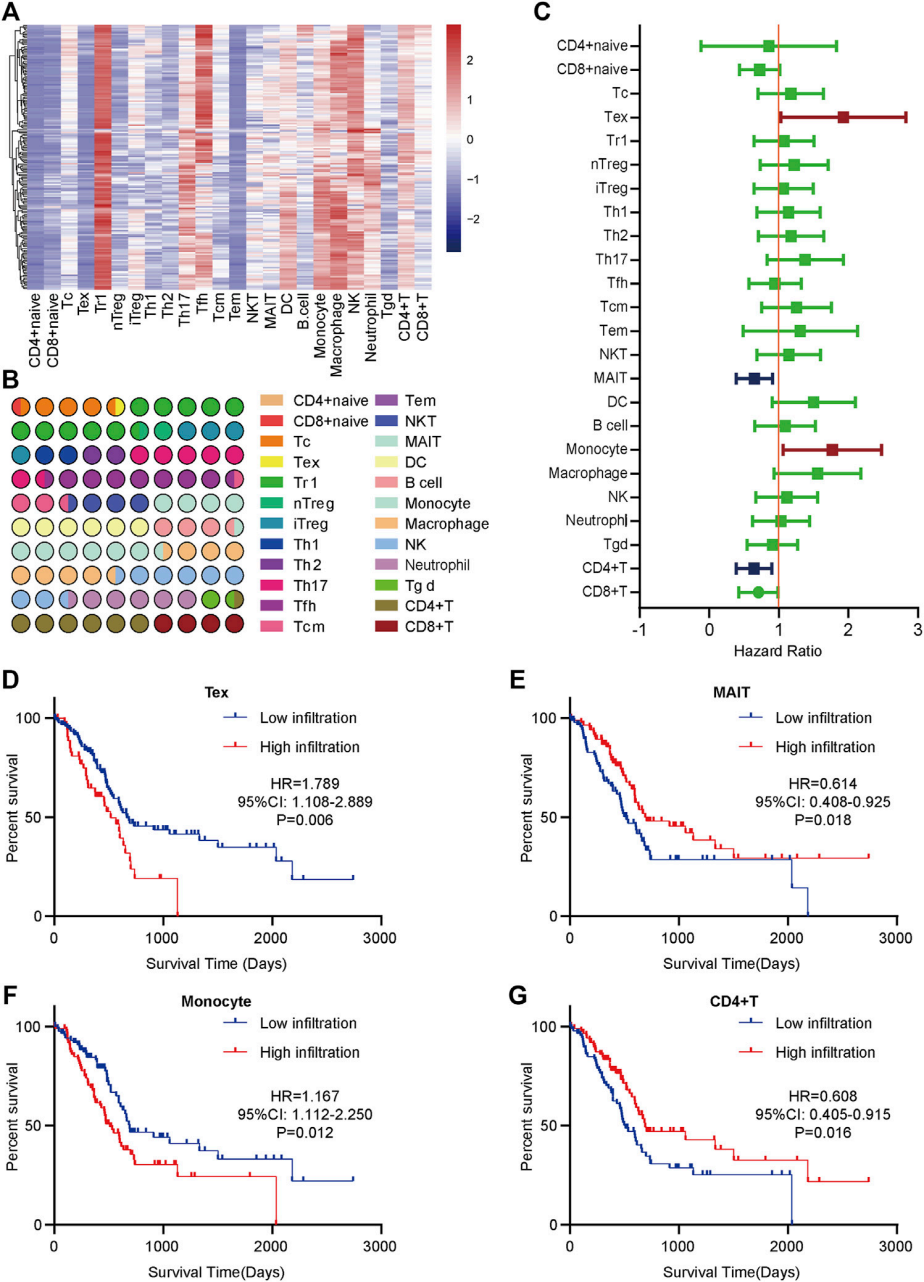
The distribution and prognostic roles of TILs in PaCa. **(A)** The heatmap of the 24 immune cells abundance based on the TCGA data. **(B)** The proportion of 24 immune cells in PaCa. **(C)** Overview of Kaplan-Meier analysis of the prognostic values of 24 selected TILs in PaCa. **(D)** High Tex infiltration predicted worse prognosis in PaCa patients. **(E)** Low MAIT infiltration predicted worse prognosis in PaCa patients. **(F)** High monocyte infiltration predicted worse prognosis in PaCa patients. **(G)** Low CD4^+^ T cell infiltration predicted worse prognosis in PaCa patients.

To obtain a systematical insight into TILs in PaCa, the ImmuCellAI tool was applied to calculate TILs composition in PaCa samples from the TCGA dataset. A heatmap was drawn to illustrate 24 immune cell proportions in these samples (Figure 1A). The fraction of immune cells varied significantly among different samples. Due to the limited number of adjacent non-cancer samples, we failed to compare the difference in TILs abundance in tumor and para-tumor samples. We next compared the proportion of different TILs in PaCa samples. The results showed that type 1 regulatory T cell (Tr 1), natural killer cell (NK), macrophage, *etc.* had higher abundance in PaCa, but CD4^+^ naïve cell, effector memory T cell (Tem), Tex, *etc.* had lower abundance (Figure 1B).

To assess the prognostic values of infiltrating TILs for PaCa patients, survival analysis with log-rank test was applied based on the TCGA-PaCa data. The patients were divided into two groups according to the median infiltration levels of TILs. The result showed that most TILs had no obvious prognostic values in PaCa patients (Figure 1C). However, patients with higher levels of Tex and monocyte had significantly worse OS (Figures 1D,F), while high infiltrating levels of mucosal-associated invariant T cell (MAIT) and CD4^+^ T cell predicted better prognosis in PaCa patients (Figures 1E,G). Overall, these findings suggest that several TILs may play a critical role in tumor progression and have specific prognostic values in PaCa.

### Establishment and validation of a TILs signature

**FIGURE 2:**
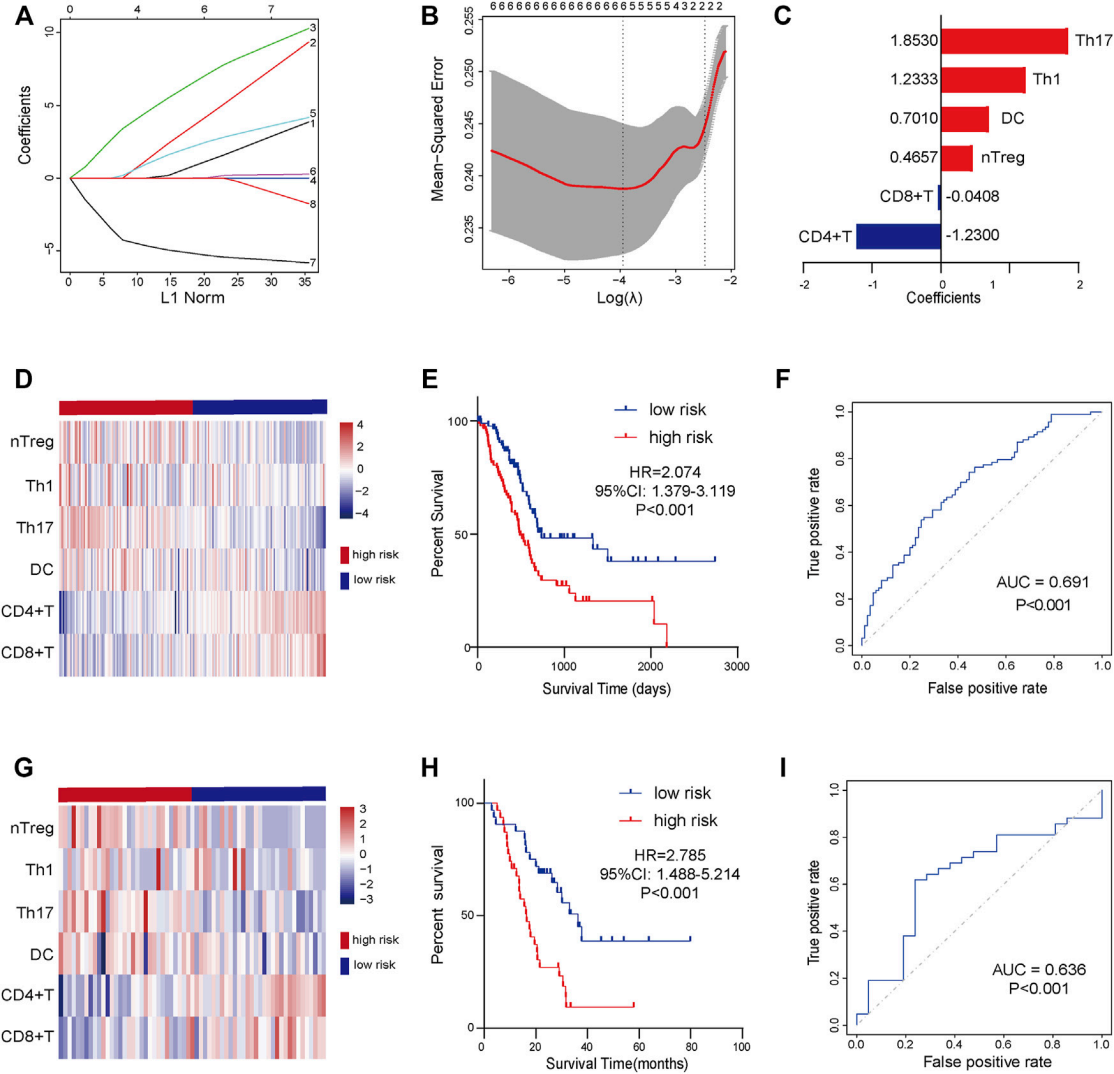
The establishment and validation of TILs-related signature in PaCa. **(A)** LASSO coefficient profiles of 8 selected TILs. **(B)** 10-fold cross-validations result which identified optimal values of the penalty parameter λ. **(C)** The distribution of LASSO Cox coefficients in the TILs-related signature. **(D)** The heatmap of six TILs infiltrating levels in the training set. **(E)** Patients in the high-risk group exhibited worse OS compared to those in the low-risk group in the training set. **(F)** ROC analysis in the training set. **(G)** The heatmap of six TILs infiltrating levels in the validated set. **(H)** Patients in the high-risk group exhibited worse OS compared to those in the low-risk group in the validated set. **(I)** ROC analysis in the validated set.

In view of the prognostic values of TILs, we next try to establish a TILs-associated prognostic signature. We first conducted univariate Cox regression to initially screen TILs with significant impacts on PaCa prognosis. A total of 8 TILs had promising prognostic impacts in PaCa (Supplementary Table S1). Subsequently, LASSO Cox analysis with ten-fold cross-validation was performed in the TCGA-PaCa dataset to further narrow the effective TILs (Figures 2A,B). Six TILs were identified and subsequently used to construct a prognostic signature. We next constructed a 6-TILs signature to assess the prognosis of PaCa patients based on the infiltrating levels of these 6 TILs and their regression coefficients (Figure 2C).

Patients in the TCGA cohort were divided into the low-risk group (*n* = 89) and the high-risk group (*n* = 89) utilizing the median risk-score as the cut-off value. The infiltrating abundance of these six TILs had an obvious distinction between the two groups (Figure 2D). The Kaplan-Meier curves exhibited that high-risk patients had notably poor prognosis in the training set (Figure 2E). The multivariate Cox analysis uncovered that this prognostic signature was an independent prognostic factor for PaCa patients (Table 2). We next conducted the ROC analysis to assess the prognostic accuracy of the risk-score, and the result showed that the risk-score had a nice prognostic accuracy (Figure 2F). Moreover, the Chi-square test showed that the risk-score was associated with several clinic-pathological features, such as age, tumor subdivision, and OS status (Table 3).

**TABLE 2:** Univariate and multivariate analysis of survival factors in patients with PaCa.

Characteristics	Univariate analysis		Multivariate analysis			
	HR	95% CI	*p*-value	HR	95% CI	*p*-value
Gender	0.81	0.54–1.22	0.320			
Age	1.03	1.01–1.05	0.010	1.01	0.99–1.04	0.271
Subdivision	0.92	0.69–1.24	0.585			
Grade	1.45	1.09–1.93	0.010	0.77	0.51–1.18	0.230
T stage	1.60	1.00–2.40	0.046	0.98	0.59–1.63	0.940
N stage	2.16	1.29–3.63	0.004	1.86	1.08–3.19	0.025
Clinical stage	1.20	0.84–1.74	0.319			
Risk-score	66.20	12.93–338.95	<0.001	30.48	5.19–179.22	<0.001

**TABLE 3:** Association between risk-score and patients’ characteristics in PaCa.

Characteristics	Cases	Low-risk		High-risk		χ^2^	*p*-value
		Cases	Proportion (%)	Cases	Proportion (%)		
**Gender**						0.363	0.547
Female	80	42	52.50	38	47.50		
Male	98	47	47.96	51	52.04		
Age						7.327	0.007
≤60	59	38	64.41	21	35.59		
>60	119	51	42.86	68	57.14		
**Subdivision**						10.916	0.012
Body	14	6	42.86	8	57.14		
Head	138	69	50.00	69	50.00		
Tail	15	4	26.67	11	73.33		
Other	11	10	90.91	1	9.09		
**Grade**						3.553	0.314
G1	30	18	60.00	12	40.00		
G2	96	49	51.04	47	48.96		
G3	48	22	45.83	26	54.17		
G4	2	0	0.00	2	100.00		
**T stage**						5.635	0.131
T1	7	4	57.14	3	42.86		
T2	24	17	70.83	7	29.17		
T3	142	65	45.77	77	54.23		
T4	3	1	33.33	2	66.67		
**N stage**						1.673	0.196
N0	50	21	42.00	29	58.00		
N1	123	65	52.85	58	47.15		
**TNM stage**						5.221	0.156
TNM1	21	14	66.67	7	33.33		
TNM2	146	71	48.63	75	51.37		
TNM3	4	1	25.00	3	75.00		
TNM4	5	1	20.00	4	80.00		
**OS status**						14.073	<0.001
Alive	85	55	64.71	30	35.29		
Dead	93	34	36.56	59	63.44		

To further validate the prognostic value of signature in PaCa, we next used 63 patients from the GSE57495 dataset as the validation cohort. Similar to the training cohort, the infiltrating abundance of these six TILs had an obvious distinction between the low-risk group (*n* = 32) and the high-risk group (*n* = 31) (Figure 2G). Kaplan-Meier curves suggested that the high-risk group patients had significantly worse outcomes than the low-risk group (Figure 2H), and the ROC analysis verified that the risk-score had good prognostic accuracy (Figure 2I). To sum up, our results indicated a satisfactory value of the TILs-associated signature for survival prediction.

### Identification of DEGs and enrichment analysis

**FIGURE 3:**
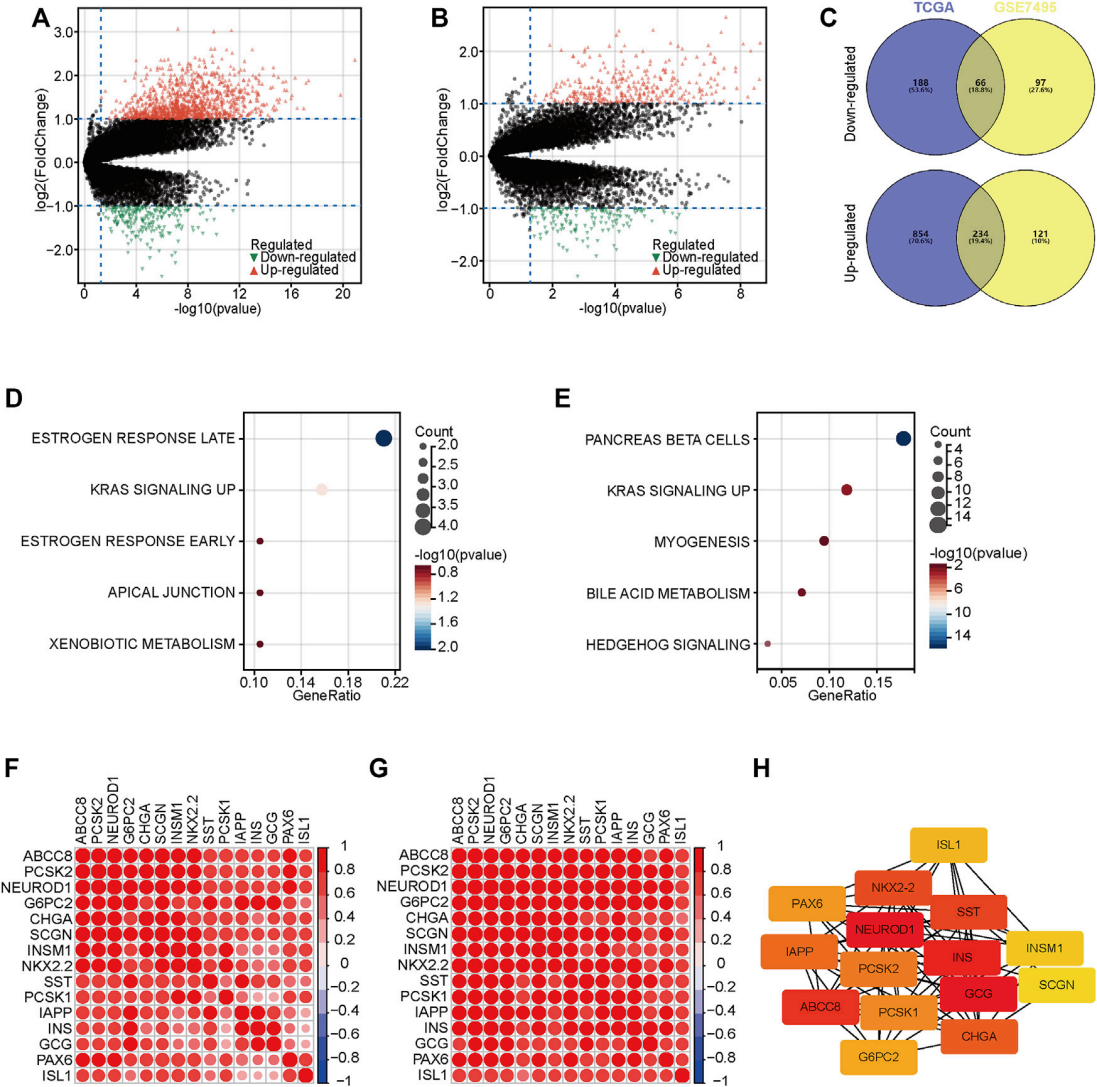
DEGs between low- and high-risk groups. **(A,B)** The volcano plot of DEGs with the thresholds of |log2[fold change (FC)]| >1 and *p* < 0.05 in the TCGA and GSE57495 datasets. © Intersection of co-DEGs between the TCGA and GSE57495 datasets. **(D,E)** Hallmark enrichment analysis of highly expressed genes in the high-risk and the low-risk groups. **(F,G)** Correlation analysis of 15 genes in the “pancreas beta cells” terms. **(H)** PPI network analysis of 15 genes in the “pancreas beta cells” terms.

To further understand the progression of PaCa, we exacted DEGs between two groups in the TCGA and GSE57495 datasets (Figures 3A,B). We next extracted co-DEGs in the TCGA and GSE57495 datasets for enrichment analysis (Figure 3C). Then, the Hallmark enrichment analysis was performed. The results showed that highly expressed genes in the high-risk groups were significantly associated with estrogen response and KRAS signaling (Figure 3D), and highly expressed genes in the low-risk groups were significantly associated with pancreas beta cells and KRAS signaling (Figure 3E). Considering enrichment numbers, we further chose genes in the “pancreas beta cells” terms for further analysis. A total of 15 genes in the “pancreas beta cells” terms were highly correlated with each other in the TCGA and GSE57495 datasets (Figures 3F,G), and the PPI network showed that NEUROD1 was the hub gene among these relative genes (Figure 3H). Taken together, we selected NEUROD1 for further investigation.

### Validation of the role of NEUROD1 in PaCa

**FIGURE 4:**
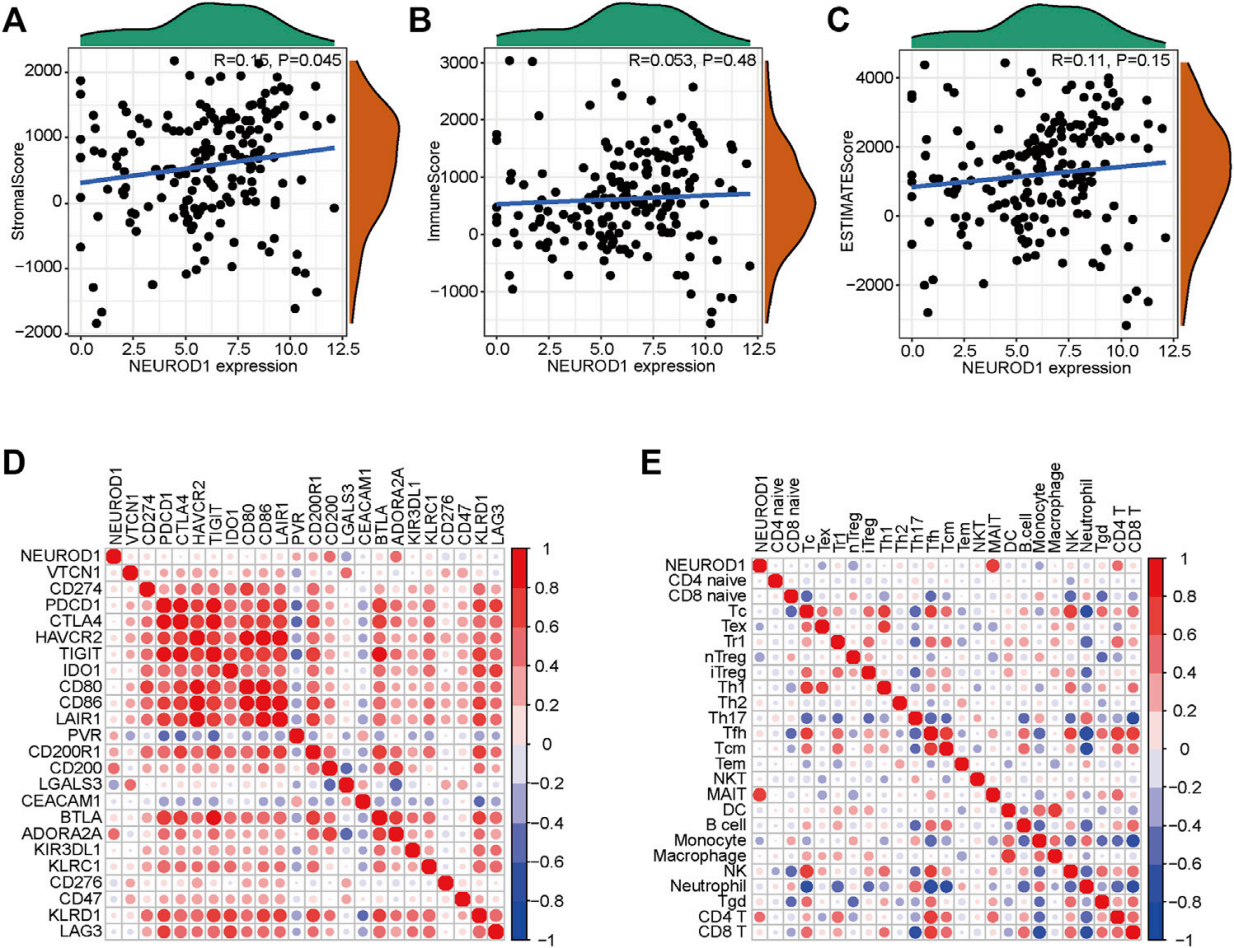
NEUROD1 was not related to anti-tumor immunity in the TCGA dataset. **(A–C)** Association between NEUROD1 expression and stromal score, immune score, and ESTIMATA score in the TCGA dataset. **(D,E)** Association between NEUROD1 expression and immune checkpoints expression as well as immune cell infiltrations in the TCGA dataset.

Given that NEUROD1 was uncovered based on the TILs-based prognostic signature, we supposed that NEUROD1 may be associated with immune cell infiltrations. However, NEUROD1 was not correlated with stromal score, immune score, and ESTIMATA score (Figures 4A–C). Similarly, NEUROD1 was not correlated with most immune checkpoints and immune cells, except MAIT and CD4^+^ T cell (Figures 4D,E). Moreover, the results from the GSE57495 dataset were also similar (Supplementary Figure S1E). Overall, NEUROD1 was not associated with anti-tumor immunity in PaCa.

**FIGURE 5:**
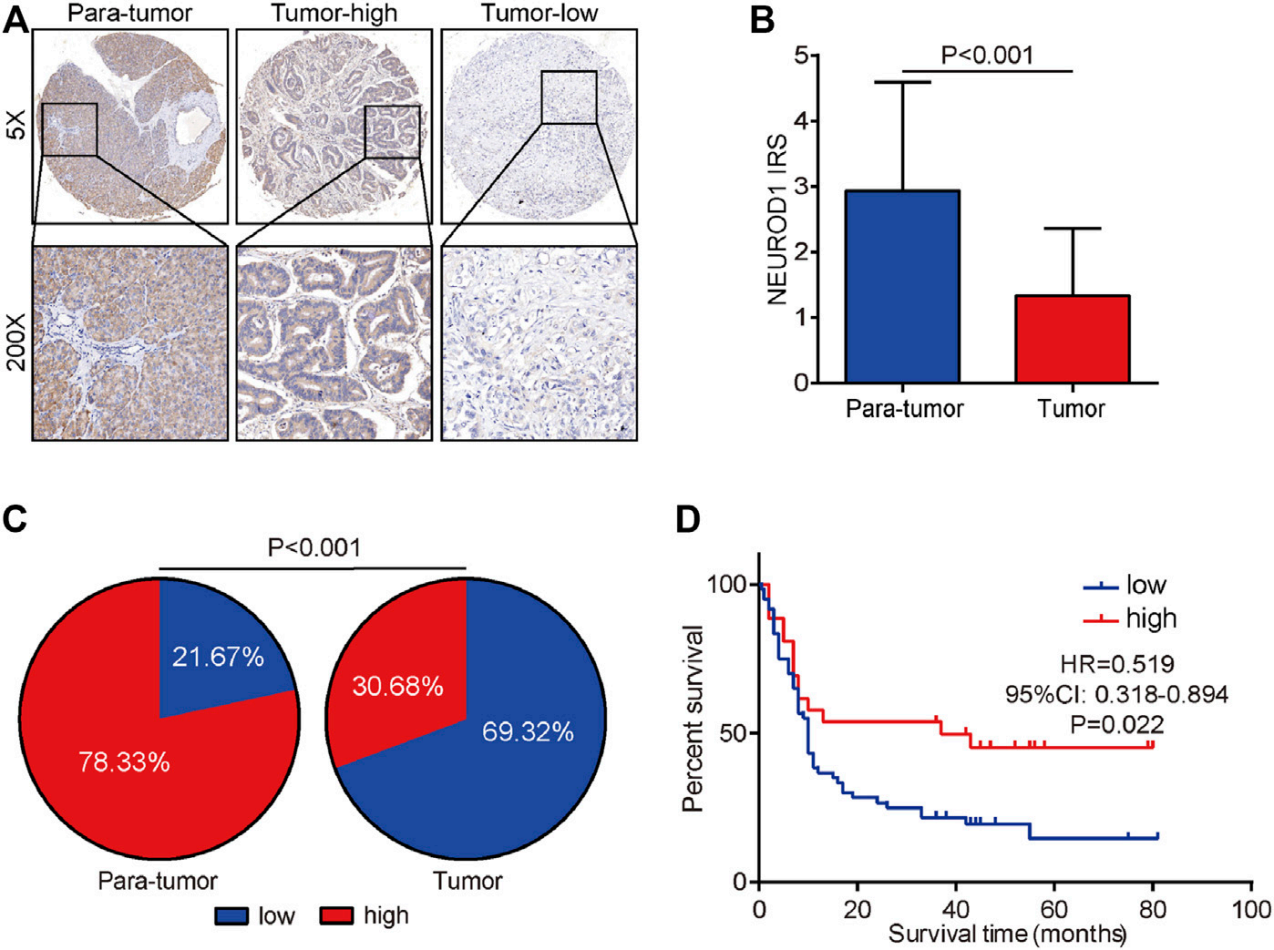
Expression and prognostic value of NEUROD1 protein in PaCa. **(A)** Representative microphotographs of NEUROD1 in tumor and para-tumor samples. Brown, NEUROD1. Blue, hematoxylin. Original magnification: ×200. Samples with NEUROD1 IRS ≥ 2 were deemed to be the high-expression group, and the others were deemed to be the low-expression group. **(B)** The expression of NEUROD1 protein in PaCa tissues and para-tumor tissues. A significant decrease in NEUROD1 expression was observed in PaCa tissues. **(C)** NEUROD1 protein expression intensity proportion of PaCa tissues and para-tumor tissues. Low expression: IRS < 2; high expression: IRS ≥2. **(D)** Kaplan-Meier analysis of NEUROD1 in the HPanA150Su01 cohort. High NEUROD1 expression predicted better prognosis in PaCa patients.

We next validated the expression and prognostic value of NEUROD1 in PaCa. NEUROD1 was significantly downregulated in tumor samples compared with para-tumor samples and most tumor samples lowly expressed NEUROD1 (Figures 5A–C). Moreover, NEUROD1 expression was notably associated with N stage and OS status (Table 4). In addition, in the HPanA150Su01 cohort, high expression of NEUROD1 was significantly correlated with better prognosis (Figure 5D). However, NEUROD1 was not an independent survival factor in PaCa (Table 5). Collectively, NEUROD1 could be used as a novel prognostic biomarker in PaCa.

**TABLE 4:** Association between NEUROD1 expression and baseline characteristics in PaCa.

Characteristics		n	NEUROD1 expression		χ^2^	*p* value
			Low	High		
Gender	Female	32	23	9	0.155	0.694
	Male	56	38	18		
Age	≤60	41	27	14	0.351	0.554
	>60	46	33	13		
	Unknown		1			
Grade	Well-differentiated	56	41	15	1.099	0.294
	Poor-differentiated	32	20	12		
T stage	T1-2	72	51	21	0.103	0.748
	T3	15	10	5		
	Unknown		1			
N stage	N0	46	27	19	4.777	0.029
	N1	37	30	7		
	Unknown		5			
TNM stage	1	37	22	15	3.057	0.080
	2	48	37	11		
	Unknown		3			
OS status	Alive	23	11	12	6.763	0.009
	Dead	65	50	15		

**TABLE 5:** Univariate and multivariate analysis of prognostic factors in PaCa.

Characteristics	Univariate analysis			Multivariate analysis		
	HR	95%CI	*p* value	HR	95%CI	*p* value
Gender (male vs. female)	1.13	0.68–1.88	0.635			
Age (>60 vs. ≤60)	1.17	0.72–1.91	0.521			
Grade (poor vs. well-differentiated)	2.06	1.25–3.40	0.004	2.92	1.66–5.15	<0.001
T stage (T3 vs. T1-2)	0.91	0.46–1.79	0.791			
N stage (N1 vs. N0)	1.78	1.07–2.96	0.027	1.95	0.73–5.16	0.181
TNM stage (2 vs. 1)	1.81	1.08–3.05	0.026	1.39	0.52–3.67	0.512
NEUROD1 (high vs. low expression)	0.79	0.55–1.11	0.173			

## Discussion

Infiltrating immune cells in the tumor microenvironment have been uncovered to play prominent and respective roles in the biological behaviors of various cancers (Mattei et al., 2012; Miyazaki et al., 2018; Georgouli et al., 2019). For example, high infiltration of CD8^+^ T cells usually inhibited cancer progression and indicated better prognosis in most cancers, but was associated with poor prognosis in clear cell renal cell carcinoma (Qi et al., 2020; Wu et al., 2021). It has been revealed that identifying subtypes of the immune microenvironment in PaCa provides promising opportunities for therapeutic development based on the personalization of systemic immunotherapies (Danilova et al., 2019). Thus, summarizing the features of immune cells in the tumor microenvironment is essential for risk classification, treatment, and prognosis assessment for PaCa. In the current study, we assessed infiltrating levels of 24 immune cells and explored their potential prognostic values in PaCa through the ImmuCellAI algorithm.

The TILs are mainly composed of B cells, T cells, macrophages, monocytes, NK cells, etc., which act as significant roles in promoting and/or suppressing cancer progression (McAllister and Weinberg, 2014; Pushalkar et al., 2018). B cells, producers of antibodies, not only are significant components of the adaptive immune system, but also could co-operate with other TILs by secreting cytokine and presenting antigens (Gupta et al., 2019). T cells contain a large family, including CD8^+^ T cells, CD4^+^ T cells, Th cells, Treg cells, and MAITs. Most T cell subsets play critical roles in tumor control (Vigano et al., 2019), but several kinds of T cells can also promote cancer progression. For example, as a member of CD8^+^ T cell subsets, Tex loses robust effector functions by expressing multiple inhibitors, which are defined by alterant transcriptional programs (Wherry and Kurachi, 2015). A previous study uncovered that high level of CD8^+^ Tex expressing PD1 predicted worse clinical outcomes in hepatocellular carcinoma (Ma et al., 2019). Multiply studies have demonstrated that infiltrating abundance of specific TILs is associated with improved or unfavorable clinical outcomes in different cancers (Stanton and Disis, 2016; Liu et al., 2017; Bubie et al., 2020). In this study, we found that high infiltrating levels of Tex and monocyte predicted worse OS, while low abundance of MAIT and CD4^+^ T cell were associated with poor prognosis in PaCa patients, suggesting several types of TILs had encouraging prognostic impacts on PaCa.

As well known, PaCa has been identified as a type of aggressive malignancy with awfully poor prognosis. Growing numbers of studies attempt to systematically summarize the malignant characterization of PaCa and develop prognostic risk identifiers from the multi-omics perspective (Thomas et al., 2014; Bailey et al., 2016). Besides, immune-related genes are also treated as hotspots in the field of prognostic assessment. Zhang *et al.* developed a PD-L2-based immune signature to exactly predict survival in resected PaCa (Zhang et al., 2019). Meng *et al.* screened DEGs between high and low immune score groups, and further established an 8-mRNA signature prognostic identifier for PaCa (Meng et al., 2020). However, as far as we know, no exploration of prognostic values of combinations of multiple immune cells has been conducted in cancers to date. In this report, we developed a TILs-related prognostic signature, consisting of Th17, Th1, DC, nTreg, CD8^+^ T cell, and CD4^+^ T cell, which could precisely indicate prognosis in PaCa patients. These findings suggested that different TILs combinations might obtain better predictive values in prognostic assessment for cancerous diseases.

Additionally, we screened the risk-related DEGs and identified NEUROD1 as a novel biomarker in PaCa. NEUROD1 has been identified as a subtype marker in neuroendocrine tumors, especially small-cell lung cancer (Dora et al., 2022). However, NEUROD1 is also revealed to express in other tumor cells and play a significant role in tumor progression. For example, NEUROD1 promotes neuroblastoma progression and is mediated by LINC00839/miR-454-3p axis (Zhang et al., 2022). In addition, NEUROD1 promotes tumor cell proliferation and tumorigenesis by directly activating the pentose phosphate pathway in colorectal cancer (Li et al., 2021). In addition to regulating the tumor progression, NEUROD1 could be also a drug target in cancer therapy. In small-cell lung cancer, NEUROD1-dependent genes are specific targets of lurbinectedin (Costanzo et al., 2022). Moreover, NEUROD1 could be used as a marker for efficacy monitoring as well. Breast cancer patients with positive serum pretreatment NEUROD1 methylation exhibited poor prognosis (Fiegl et al., 2008). In this research, we found NEUROD1 was associated with better prognosis in PaCa, but its functional role still needed to be explored.

## Conclusion

To sum up, we analyzed the 24 TIL subgroups in PaCa samples based on transcriptome data using the ImmuCellAI tool. As an important result, we identified and validated a six-TILs prognostic signature, which could precisely predict prognosis in PaCa patients. Moreover, based on the novel signature, we also identified NEUROD1 as a novel biomarker in PaCa. However, the findings of the current research should be further validated using large-scale clinical cohorts.

## Data Availability

The datasets presented in this study can be found in online repositories. The names of the repository/repositories and accession number(s) can be found in the article/Supplementary Material. Some preliminary results have already been preprinted online in bioRxiv, doi: https://doi.org/10.1101/2020.03.30.017327.

## Abbreviations

PaCa, pancreatic cancer; TIL, tumor-infiltrating lymphocyte; RNA-seq, RNA sequencing; Treg, regulatory cell; Tc, cytotoxic T cell; Tex, exhausted T cell; Th1, T helper 1 cell; DC, dendritic cell; OS, overall survival; LASSO, least absolute shrinkage and selection operator; ROC, receiver operating characteristic; DEG, differentially expressed gene; TMA, tissue microarray; IHC, immunohistochemistry; IRS, immunoreactivity score; HR, hazard ratio; NK, natural killer cell; Tem, effector memory T cell; MAIT, mucosal-associated invariant T cell.

## References

[B1] Aran D. Hu Z. Butte A. J. (2017). Xcell: Digitally portraying the tissue cellular heterogeneity landscape. Genome Biol. 18 (1), 220. Epub 2017/11/17. 10.1186/s13059-017-1349-1 29141660 PMC5688663

[B2] Bailey P. Chang D. K. Nones K. Johns A. L. Patch A. M. Gingras M. C. (2016). Genomic analyses identify molecular subtypes of pancreatic cancer. Nature 531 (7592), 47–52. Epub 2016/02/26. 10.1038/nature16965 26909576

[B3] Becht E. Giraldo N. A. Lacroix L. Buttard B. Elarouci N. Petitprez F. (2016). Estimating the population abundance of tissue-infiltrating immune and stromal cell populations using gene expression. Genome Biol. 17 (1), 218. 10.1186/s13059-016-1070-5 27765066 PMC5073889

[B4] Bubie A. Gonzalez-Kozlova E. Akers N. Villanueva A. Losic B. (2020). Tumor fitness, immune exhaustion and clinical outcomes: Impact of immune checkpoint inhibitors. Sci. Rep. 10 (1), 5062. Epub 2020/03/21. 10.1038/s41598-020-61992-2 32193450 PMC7081289

[B5] Cai Y. Ji W. Sun C. Xu R. Chen X. Deng Y. (2021). Interferon-induced transmembrane protein 3 shapes an inflamed tumor microenvironment and identifies immuno-hot tumors. Front. Immunol. 12, 704965. Epub 2021/08/31. 10.3389/fimmu.2021.704965 34456915 PMC8385493

[B6] Chen D. T. Davis-Yadley A. H. Huang P. Y. Husain K. Centeno B. A. Permuth-Wey J. (2015). Prognostic fifteen-gene signature for early stage pancreatic ductal adenocarcinoma. PLoS One 10 (8), e0133562. Epub 2015/08/08. 10.1371/journal.pone.0133562 26247463 PMC4527782

[B7] Costanzo F. Martinez Diez M. Santamaria Nunez G. Diaz-Hernandez J. I. Genes Robles C. M. Diez Perez J. (2022). Promoters of Ascl1- and neurod1-dependent genes are specific targets of lurbinectedin in sclc cells. EMBO Mol. Med. 14 (4), e14841. Epub 2022/03/10. 10.15252/emmm.202114841 35263037 PMC8988166

[B8] Danilova L. Ho W. J. Zhu Q. Vithayathil T. De Jesus-Acosta A. Azad N. S. (2019). Programmed cell death ligand-1 (Pd-L1) and Cd8 expression profiling identify an immunologic subtype of pancreatic ductal adenocarcinomas with favorable survival. Cancer Immunol. Res. 7 (6), 886–895. Epub 2019/05/03. 10.1158/2326-6066.CIR-18-0822 31043417 PMC6548624

[B9] Dora D. Rivard C. Yu H. Pickard S. L. Laszlo V. Harko T. (2022). Protein expression of immune checkpoints sting and mhcii in small cell lung cancer. Cancer Immunol. Immunother. Epub 2022/08/18. 10.1007/s00262-022-03270-w PMC10991160 35978199

[B10] Dougan S. K. (2017). The pancreatic cancer microenvironment. Cancer J. 23 (6), 321–325. Epub 2017/12/01. 10.1097/PPO.0000000000000288 29189327

[B11] Fiegl H. Jones A. Hauser-Kronberger C. Hutarew G. Reitsamer R. Jones R. L. (2008). Methylated Neurod1 promoter is a marker for chemosensitivity in breast cancer. Clin. Cancer Res. 14 (11), 3494–3502. Epub 2008/06/04. 10.1158/1078-0432.CCR-07-4557 18519782

[B12] Foley K. Kim V. Jaffee E. Zheng L. (2016). Current progress in immunotherapy for pancreatic cancer. Cancer Lett. 381 (1), 244–251. Epub 2016/01/03. 10.1016/j.canlet.2015.12.020 26723878 PMC4919239

[B13] Georgouli M. Herraiz C. Crosas-Molist E. Fanshawe B. Maiques O. Perdrix A. (2019). Regional activation of myosin ii in cancer cells drives tumor progression via a secretory cross-talk with the immune microenvironment. Cell 176 (4), 757–774. e23Epub 2019/02/05. 10.1016/j.cell.2018.12.038 30712866 PMC6370915

[B14] Gupta P. Chen C. Chaluvally-Raghavan P. Pradeep S. (2019). B cells as an immune-regulatory signature in ovarian cancer. Cancers (Basel) 11 (7), E894. Epub 2019/06/30. 10.3390/cancers11070894 31248034 PMC6678944

[B15] Li T. Fan J. Wang B. Traugh N. Chen Q. Liu J. S. (2017). Timer: A web server for comprehensive analysis of tumor-infiltrating immune cells. Cancer Res. 77 (21), e108–e110. Epub 2017/11/03. 10.1158/0008-5472.CAN-17-0307 29092952 PMC6042652

[B16] Li Z. He Y. Li Y. Li J. Zhao H. Song G. (2021). Neurod1 promotes tumor cell proliferation and tumorigenesis by directly activating the pentose phosphate pathway in colorectal carcinoma. Oncogene 40 (50), 6736–6747. Epub 2021/10/18. 10.1038/s41388-021-02063-2 34657129

[B17] Liberzon A. Subramanian A. Pinchback R. Thorvaldsdottir H. Tamayo P. Mesirov J. P. (2011). Molecular signatures database (msigdb) 3.0. Bioinformatics 27 (12), 1739–1740. 10.1093/bioinformatics/btr260 21546393 PMC3106198

[B18] Liu X. Wu S. Yang Y. Zhao M. Zhu G. Hou Z. (2017). The prognostic landscape of tumor-infiltrating immune cell and immunomodulators in lung cancer. Biomed. Pharmacother. 95, 55–61. Epub 2017/08/22. 10.1016/j.biopha.2017.08.003 28826097

[B19] Ma J. Zheng B. Goswami S. Meng L. Zhang D. Cao C. (2019). Pd1(Hi) Cd8(+) T cells correlate with exhausted signature and poor clinical outcome in hepatocellular carcinoma. J. Immunother. Cancer 7 (1), 331. Epub 2019/12/01. 10.1186/s40425-019-0814-7 31783783 PMC6884778

[B20] Mattei F. Schiavoni G. Sestili P. Spadaro F. Fragale A. Sistigu A. (2012). Irf-8 controls melanoma progression by regulating the cross talk between cancer and immune cells within the tumor microenvironment. Neoplasia 14 (12), 1223–1235. Epub 2013/01/12. 10.1593/neo.121444 23308054 PMC3540947

[B21] McAllister S. S. Weinberg R. A. (2014). The tumour-induced systemic environment as a critical regulator of cancer progression and metastasis. Nat. Cell Biol. 16 (8), 717–727. Epub 2014/08/02. 10.1038/ncb3015 25082194 PMC6220424

[B22] Mei J. Liu Y. Yu X. Hao L. Ma T. Zhan Q. (2021). Ywhaz interacts with Daam1 to promote cell migration in breast cancer. Cell Death Discov. 7 (1), 221. Epub 2021/08/29. 10.1038/s41420-021-00609-7 34453038 PMC8397740

[B23] Mei J. Xu B. Hao L. Xiao Z. Liu Y. Yan T. (2020). Overexpressed Daam1 correlates with metastasis and predicts poor prognosis in breast cancer. Pathol. Res. Pract. 216 (3), 152736. Epub 2019/11/24. 10.1016/j.prp.2019.152736 31757662

[B24] Meng Z. Ren D. Zhang K. Zhao J. Jin X. Wu H. (2020). Using estimate algorithm to establish an 8-mrna signature prognosis prediction system and identify immunocyte infiltration-related genes in pancreatic adenocarcinoma. Aging Albany N. Y., 12. 5048. Epub 2020/03/18. 10.18632/aging.102931 PMC7138590 32181755

[B25] Miao Y. R. Zhang Q. Lei Q. Luo M. Xie G. Y. Wang H. (2020). Immucellai: A unique method for comprehensive T‐cell subsets abundance prediction and its application in cancer immunotherapy. Adv. Sci. 7, 1902880. 10.1002/advs.201902880 PMC7141005 32274301

[B26] Miyazaki T. Ikeda K. Sato W. Horie-Inoue K. Inoue S. (2018). Extracellular vesicle-mediated Ebag9 transfer from cancer cells to tumor microenvironment promotes immune escape and tumor progression. Oncogenesis 7 (1), 7. Epub 2018/01/25. 10.1038/s41389-017-0022-6 29362448 PMC5833691

[B27] Newman A. M. Liu C. L. Green M. R. Gentles A. J. Feng W. Xu Y. (2015). Robust enumeration of cell subsets from tissue expression profiles. Nat. Methods 12 (5), 453–457. Epub 2015/03/31. 10.1038/nmeth.3337 25822800 PMC4739640

[B28] Pushalkar S. Hundeyin M. Daley D. Zambirinis C. P. Kurz E. Mishra A. (2018). The pancreatic cancer microbiome promotes oncogenesis by induction of innate and adaptive immune suppression. Cancer Discov. 8 (4), 403–416. Epub 2018/03/24. 10.1158/2159-8290.CD-17-1134 29567829 PMC6225783

[B29] Qi Y. Xia Y. Lin Z. Qu Y. Qi Y. Chen Y. (2020). Tumor-infiltrating Cd39(+)Cd8(+) T cells determine poor prognosis and immune evasion in clear cell renal cell carcinoma patients. Cancer Immunol. Immunother. 69 (8), 1565–1576. Epub 2020/04/20. 10.1007/s00262-020-02563-2 32306075 PMC11027701

[B30] Racle J. de Jonge K. Baumgaertner P. Speiser D. E. Gfeller D. (2017). Simultaneous enumeration of cancer and immune cell types from bulk tumor gene expression data. Elife 6, e26476. Epub 2017/11/14. 10.7554/eLife.26476 29130882 PMC5718706

[B31] Shen W. Song Z. Zhong X. Huang M. Shen D. Gao P. (2022). Sangerbox: A comprehensive, interaction-friendly clinical bioinformatics analysis platform. iMeta 1 (3), e36. 10.1002/imt2.36 PMC10989974 38868713

[B32] Siegel R. L. Miller K. D. Fuchs H. E. Jemal A. (2022). Cancer statistics, 2022. Ca. Cancer J. Clin. 72 (1), 7–33. Epub 2022/01/13. 10.3322/caac.21708 35020204

[B33] Stanton S. E. Disis M. L. (2016). Clinical significance of tumor-infiltrating lymphocytes in breast cancer. J. Immunother. Cancer 4, 59. Epub 2016/10/26. 10.1186/s40425-016-0165-6 27777769 PMC5067916

[B34] Stenzel P. J. Schindeldecker M. Tagscherer K. E. Foersch S. Herpel E. Hohenfellner M. (2020). Prognostic and predictive value of tumor-infiltrating leukocytes and of immune checkpoint molecules Pd1 and Pdl1 in clear cell renal cell carcinoma. Transl. Oncol. 13 (2), 336–345. Epub 2019/12/28. 10.1016/j.tranon.2019.11.002 31881506 PMC7031108

[B35] Tahkola K. Mecklin J. P. Wirta E. V. Ahtiainen M. Helminen O. Bohm J. (2018). High immune cell score predicts improved survival in pancreatic cancer. Virchows Arch. 472 (4), 653–665. Epub 2018/01/23. 10.1007/s00428-018-2297-1 29356891

[B36] Thomas J. K. Kim M. S. Balakrishnan L. Nanjappa V. Raju R. Marimuthu A. (2014). Pancreatic cancer database: An integrative resource for pancreatic cancer. Cancer Biol. Ther. 15 (8), 963–967. Epub 2014/05/21. 10.4161/cbt.29188 24839966 PMC4119079

[B37] Vigano S. Alatzoglou D. Irving M. Menetrier-Caux C. Caux C. Romero P. (2019). Targeting adenosine in cancer immunotherapy to enhance T-cell function. Front. Immunol. 10, 925. Epub 2019/06/28. 10.3389/fimmu.2019.00925 31244820 PMC6562565

[B38] Wherry E. J. Kurachi M. (2015). Molecular and cellular insights into T cell exhaustion. Nat. Rev. Immunol. 15 (8), 486–499. Epub 2015/07/25. 10.1038/nri3862 26205583 PMC4889009

[B39] Winer A. Dotan E. (2021). Treatment paradigms for older adults with pancreatic cancer: A nuanced approach. Curr. Treat. Options Oncol. 22 (11), 104. Epub 2021/10/02. 10.1007/s11864-021-00892-7 34596801

[B40] Wu X. Jiang D. Liu H. Lu X. Lv D. Liang L. (2021). Cd8(+) T cell-based molecular classification with heterogeneous immunogenomic landscapes and clinical significance of clear cell renal cell carcinoma. Front. Immunol. 12, 745945. Epub 2022/01/01. 10.3389/fimmu.2021.745945 34970257 PMC8713701

[B41] Zhang Q. Wei J. Li N. Liu B. (2022). Linc00839 promotes neuroblastoma progression by sponging mir-454-3p to up-regulate Neurod1. Neurochem. Res. 47 (8), 2278–2293. Epub 2022/05/24. 10.1007/s11064-022-03613-0 35606572

[B42] Zhang Y. Xu J. Hua J. Liu J. Liang C. Meng Q. (2019). A Pd-L2-Based immune marker signature helps to predict survival in resected pancreatic ductal adenocarcinoma. J. Immunother. Cancer 7 (1), 233. Epub 2019/08/30. 10.1186/s40425-019-0703-0 31464648 PMC6716876

