# Supplementary material for: NEUROD1 predicts better prognosis in pancreatic cancer revealed by a TILs-based prognostic signature
Source: Front Pharmacol. 2022 Oct 13;13:1025921. doi: 10.3389/fphar.2022.1025921 (PMC9612957; doi:10.3389/fphar.2022.1025921)
Supplement: Supplementary file 1 [file DataSheet1.DOCX]

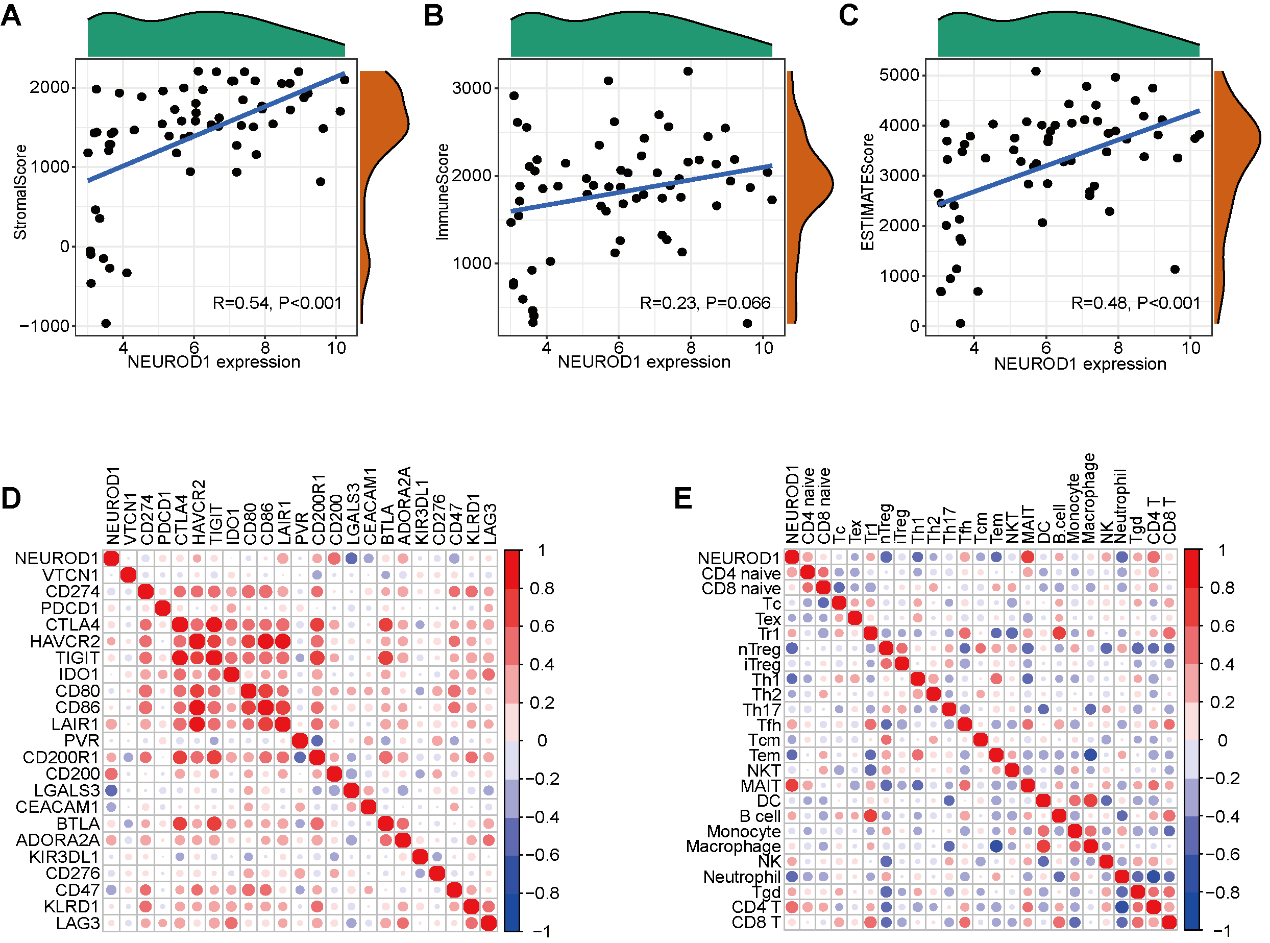


**Figure S1. NEUROD1 was not related to anti-tumor immunity in the GSE57495 dataset**

(A, B, C) Association between NEUROD1 expression and stromal score, immune score, and ESTIMATA score in the GSE57495 dataset. (D, E) Association between NEUROD1 expression and immune checkpoints expression as well as immune cell infiltrations in the GSE57495 dataset.

**Table S1. Univariate Cox regression analysis of TIICs infiltration in PAAD.**

| **TIICs** | **Beta** | **HR (95%CI for HR)** | **P-value** |
| --- | --- | --- | --- |
| CD4.naive | -320 | 1.5e-140 (0-Inf) | 0.750 |
| CD8.naive | -7 | 0.00089 (3.9e-08-20) | 0.170 |
| Tc | 3.3 | 26 (0.48-1400) | 0.110 |
| Tex | 11 | 70000 (0.65-7.6e+09) | 0.059 |
| Tr1 | -0.37 | 0.69 (0.015-32) | 0.850 |
| nTreg | 6.6 | 730 (1.4-380000) | **0.039** |
| iTreg | -0.71 | 0.49 (0.021-12) | 0.660 |
| Th1 | 4.5 | 88 (1.3-6100) | **0.038** |
| Th2 | -0.99 | 0.37 (0.00052-270) | 0.770 |
| Th17 | 5.6 | 280 (3.6-22000) | **0.011** |
| Tfh | -0.29 | 0.75 (0.13-4.2) | 0.740 |
| Tcm | 2.2 | 9.3 (0.14-630) | 0.300 |
| Tem | -8.9 | 0.00014 (3.7e-16-5.3e+07) | 0.510 |
| NKT | 1.1 | 3 (0.067-140) | 0.570 |
| MAIT | -4.1 | 0.017 (0.00045-0.64) | **0.028** |
| DC | 4.7 | 110 (3.2-3500) | **0.009** |
| B.cell | -1.2 | 0.31 (0.0067-14) | 0.550 |
| Monocyte | 5.3 | 210 (3.5-13000) | **0.011** |
| Macrophage | 1.8 | 5.9 (0.56-61) | 0.140 |
| NK | -0.057 | 0.94 (0.053-17) | 0.970 |
| Neutrophil | -1.6 | 0.2 (0.0031-13) | 0.450 |
| Tgd | -2.7 | 0.07 (0.00018-28) | 0.380 |
| CD4.T | -5.2 | 0.0056 (0.00013-0.24) | **0.007** |
| CD8.T | -4.5 | 0.011 (0.00014-0.87) | **0.043** |
